# Preclinical Evaluation of 4-Methylthiobutyl Isothiocyanate on Liver Cancer and Cancer Stem Cells with Different p53 Status

**DOI:** 10.1371/journal.pone.0070846

**Published:** 2013-08-02

**Authors:** Evelyn Lamy, Anke Hertrampf, Corinna Herz, Julia Schüler, Miriam Erlacher, Daniela Bertele, Adekunle Bakare, Meike Wagner, Timo Weiland, Ulrich Lauer, Oliver Drognitz, Roman Huber, Sascha Rohn, Torsten Giesemann, Volker Mersch-Sundermann

**Affiliations:** 1 Department of Environmental Health Sciences, Freiburg University Medical Center, Freiburg, Germany; 2 Faculty of Biology, University of Freiburg, Freiburg, Germany; 3 Department of Experimental Oncology, Oncotest GmbH, Freiburg, Germany; 4 Department of Paediatrics and Adolescent Medicine, Freiburg University Medical Center, Freiburg, Germany; 5 Department of Zoology, University of Ibadan, Ibadan, Nigeria; 6 Department of Translational Oncology, Mainz University Medical Center, Mainz, Germany; 7 Department of Internal Medicine I, Medical University Hospital Tübingen, Tübingen, Germany; 8 Department of Surgery, Freiburg University Medical Center, Freiburg, Germany; 9 Institute for Food Chemistry, University of Hamburg, Hamburg, Germany; University College London, United Kingdom

## Abstract

Isothiocyanates from plants of the order *Brassicales* are considered promising cancer chemotherapeutic phytochemicals. However, their selective cytotoxicity on liver cancer has been barely researched. Therefore, in the present study, we systematically studied the chemotherapeutic potency of 4-methylthiobutyl isothiocyanate (MTBITC). Selective toxicity was investigated by comparing its effect on liver cancer cells and their chemoresistant subpopulations to normal primary hepatocytes and liver tissue slices. Additionally, in a first assessment, the *in vivo* tolerability of MTBITC was investigated in mice. Growth arrest at G2/M and apoptosis induction was evident in all *in vitro* cancer models treated with MTBITC, including populations with cancer initiating characteristics. This was found independent from TP53; however cell death was delayed in p53 compromised cells as compared to wt-p53 cells which was probably due to differential BH3 only gene regulation i. e. Noxa and its antagonist A1. In normal hepatocytes, no apoptosis or necrosis could be detected after repeated administration of up to 50 µM MTBITC. In mice, orally applied MTBITC was well tolerated over 18 days of treatment for up to 50 mg/kg/day, the highest dose tested. In conclusion, we could show here that the killing effect of MTBITC has a definite selectivity for cancer cells over normal liver cells and its cytotoxicity even applies for chemoresistant cancer initiating cells. Our study could serve for a better understanding of the chemotherapeutic properties of isothiocyanates on human liver-derived cancer cells.

## Introduction

The hepatocellular carcinoma (HCC) is the commonest cancer of the digestive system in South East Asia and Sub-Saharan Africa; an increased incidence is also being noticed in the industrialized world [1]. The prognosis for patients with major or multifocal HCC is poor, with the 5 year survival rate being less than 5% [2]. This is mainly due to non-responsiveness to chemotherapy and radiotherapy in the treatment of HCC and impaired TP53 function has been identified as important factor for this [3]. TP53 is a key player in growth arrest and apoptosis [4] and one of the most commonly mutated tumor suppressor genes in HCC [5]. Additionally, the concept that highly treatment-resistant cancer stem cells (tumor-initiating cells, TIC) play a central role in the pathogenesis of HCC has recently captured much attention. TIC are capable of self-renewing, differentiating, and maintaining tumor growth and heterogeneity. Common anticancer treatments such as radiation and chemotherapy do not eradicate the majority of highly resistant TIC [6]. Thus, searching for alternative therapy strategies which effectively affect these subpopulations, thereby overcoming tumor resistance and do not rely upon intact p53 for cancer cell killing is of utmost importance [7].

Isothiocyanates (ITC) from plants of the order *Brassicales* are currently of great interest because of their potential application in the prevention and treatment of cancer. Numerous investigations show that naturally occurring ITC and their synthetic analogues retard or inhibit tumor cell growth, both *in vitro* and *in vivo* [8], [9]. More importantly, it was shown recently that ITC can suppress aldehyde dehydrogenase (ALDH)-positive TIC of the breast [10] and prostate [11]. However, efficacy of ITC against TIC of other organs, including the liver, is yet to be determined. In general, information on the cytotoxic and cytostatic potential of ITC on tumor cells of the liver is scarce [12]–[14]. It is a precondition that potential therapeutic agents exhibit low toxicity to normal tumor surrounding tissue, yet only very limited information exists about the effects of ITC on healthy tissues. Here we describe the antineoplastic activity of 4-methylthiobutyl isothiocyanate (MTBITC, erucin) and its selective killing of tumor cells and TIC through an p53-independent mechanism. MTBITC is obtained from enzymatic hydrolysis of glucoerucin, isolated from rocket plant species (*Eruca sativa Mill*. and *Diplotaxis tenuifolia L*.). Furthermore, it is derived *in vivo* by metabolic reduction of the isothiocyanate sulforaphane, which is characteristic of broccoli (*Brassica oleracea* L.). For our studies, we used a set of *in vitro* models consisting of HCC cell lines, chemoresistant TIC, primary normal hepatocytes and precision-cut liver tissue slices (PCLS) derived from patients to study cancer selective cytotoxicity of MTBITC. Our findings were then further substantiated by mechanistic studies on differential TP53 pathway activation upon MTBITC treatment. Based on our *in vitro* results we finally investigated the tolerability of MTBITC in a mouse model.

## Materials and Methods

### Ethical Statement

Normal hepatocytes were obtained from patients after their written informed consent from the Dept. of Surgery, Freiburg University Medical Center, Germany. This part was approved by the ethics committee of the University of Freiburg (Ethik-Kommission der Albert-Ludwigs-Universität Freiburg/Ethic commission of the Albert-Ludwigs-Universität Freiburg). For tissue slicing experiments, human liver and liver tumor resectates were obtained from patients after their written informed consent from the Dept. of General, Visceral & Transplant Surgery, University Hospital, Tübingen, Germany. The study protocol was approved by the local Ethics Committee (Ethik-Kommission an der Medizinischen Fakultät der Eberhard-Karls-Universität und am Universitätsklinikum Tübingen/Ethic commission of the medical faculty of the Eberhard-Karls-University and the University Clinic Hospital Tuebingen). Animal experiments were conducted according to the guidelines of the German Animal Welfare Act (Tierschutzgesetz) and under the permission numbers of the Regierungspräsidium Freiburg, Germany G-10/05 and 35-9185.64/1. Animal health was examined prior to randomization to ensure that only animals without any symptoms of disease were selected to enter testing procedures. During the experiments, animals were monitored twice daily regarding general condition, food and water supply.

### Chemicals

DMSO, verapamil-hydrochloride, dexamethasone, erysoline, ethanol, propidium iodide (PI) and neutral red (NR) were acquired from Sigma Aldrich (Steinheim, Germany). Dulbeccos Minimal Essential Medium (DMEM), fetal calf serum (FCS), trypsin 10×(25 mg/ml), trypsin-EDTA 10× (5 mg/ml), Hanks balanced salt buffer (HBSS, without Ca and Mg) and phosphate buffered saline (PBS, without Ca and Mg) were from PAA Laboratories GmBH (Coelbe, Germany). Penicillin-Streptomycin (P/S) solution and Hoechst 33342 solution (10 mg/ml), RPMI 1640, insulin-transferrin-selen (ITS), Collagenase type IV, and 5,5′,6,6′-tetrachloro-1,1′,3,3′-tetraethylbenzimidazolcarbocyanine iodide (JC-1) were from Life Technologies Invitrogen (Darmstadt, Germany), WST-1 reagent from Roche (Mannheim, Germany). Accumax was purchased from eBioscience (Frankfurt, Germany), Collagen G from Schubert & Weiss (Munich,Germany), Collagenase CLS II from Biochrom (Berlin, Germany) and EDTA from Serva Electrophoresis (Heidelberg, Germany). CaCl_2_, Glucose, EGTA, Acetic acid (purity 100%), Roti®-Phenol/Chloroform/Isoamylalkohol and Chloroform/Isoamylalkohol were acquired from Carl Roth (Karlsruhe, Germany), Camptothecin (CPT) from Tocris (Eching, Germany), Caspase 3/7 GLO reagent from Promega (Mannheim, Germany) and Triton X-100 from Merck (Mannheim, Germany). ROMPUN 2%, and xylazinchloride were purchased from Bayer (Leverkusen, Germany), ketanest 10% and ketaminiumchloride from Essex Tierarznei (München, Germany). 4-methylthiobutyl isothiocyanate (MTBITC, erucin) was synthesized by the Inst. of Organic Chemistry, University of Giessen, Germany as described before [15]. Valinomycin was purchased from Fluka, (Buchs, Switzerland). ITC were dissolved in sterile DMSO.

### HCC Cell Lines, Isolation and Cultivation of HCC Explants and Hepatocytes

#### HCC cell lines

HepG2 (wt-53) and Hep3B (null-p53) cell lines were obtained from the German Collection of Microorganisms and Cell Cultures (DSMZ), Braunschweig, Germany. Huh-7 cells (mut-p53) originally established by Nakabayashi et al. [16] were kindly provided by H. Blum (University Medical Center Freiburg, Germany). The cells were cultured in low glucose DMEM supplemented with 15% (HepG2) or 10% (Huh7, Hep3B) FCS and 1% P/S in a 5% CO_2_ atmosphere at 37°C until 70% of confluency and subsequently harvested with trypsin. Cell line verification was done by microscopically checking the morphology of the cells and performing growth curve analysis on a regular basis. Only cells in passage number four to ten were used. The cell culture was furthermore tested negative for mycoplasma contamination.

#### HCC explants

Human liver tumour engraftments (LIXF, liver cancer xenograft Freiburg), originally established by Oncotest GmbH, Germany, were grown subcutaneously in nude mice as described elsewhere [17]. Explants were sent to our laboratory immediately after surgery, avoiding cooling or other manipulations. The procedure for preparing cultures was in accordance with Patrawala *et al.* [18].

#### Primary human hepatocytes

Normal hepatocytes were isolated within 2 h. Assessment of non-tumorous liver parenchyma was performed by a senior pathologist with expertise in liver pathology. Hepatocytes were isolated using the two-step collagenase perfusion technique according to a modified protocol from Guguen-Guillouzo *et al.* [19] and Strom *et al.* [20]. The cells were then finally resuspended in RPMI-1640 supplemented with 15% FCS, 2 mM L-glutamine 1% P/S, 1% ITS, 100 nM dexamethasone and cultured in a 5% CO_2_ atmosphere at 37°C for 20 h.

#### Murine hepatocytes

Murine hepatocytes were freshly isolated with a similar perfusion technique described for human hepatocytes and cultured in a 5% CO_2_ atmosphere at 37°C for 10 h.

### Precision-cut Tissue Slicing (PCLS)

Slicing started within one hour of resection on a vibratome VT1200S (Leica, Wetzlar, Germany) as described before [21] Slices were incubated in oxygenated William’s E medium containing 25 mM glucose and 50 µg/ml gentamycin (Lonza Bioscience, Verviers, Belgium) in an oxygenated atmosphere (80% oxygen, 5% CO_2_).

### Determination of Drug Effect in Cancer Cells

For the experiments, cancer cells were seeded, supplemented with culture medium and incubated for 48 h at 37°C. Subsequently, the subconfluent cells were exposed to ITC for 24 h to 96 h. For exposure >24 h, the culture medium, containing ITC was replaced every 24 h.

### Detection of Cytotoxicity

#### Neutral red retention assay

The neutral red retention assay determines accumulation of the neutral red dye in lysosomes of viable, uninjured cells which was used as another parameter for the detection of cytostasis/cytotoxicity. Following compound exposure, cells were incubated for 3 h with NR dye (50 µg/ml) dissolved in the corresponding culture medium. Cells were then washed gently with prewarmed PBS and 150 µl fixation medium (EtOH:AcCOOH:deionised water, 50%:1%:49%) was added followed by gentle shaking for 20 min. Absorbance was recorded at 540 nm using a Tecan microplate reader.

### Detection of Apoptosis and Growth Arrest

#### Caspase 3/7 cleavage assay

Induction of apoptosis in cell lines and primary cells was determined by using the Caspase3/7-Glo assay (Promega, Germany) according to the manufacturer’s instructions.

Apoptosis in liver tissue slices was determined by incubation with 50 µM of *N*-acetyl-Asp-Glu-Val-Asp-aminomethylcoumarin (Ac-DEVD-AMC; Biomol, Hamburg, Germany) in assay buffer (50 mM HEPES, pH 7.4, 1% sucrose, 0.1% CHAPS, 10 mM DTT). The substrate cleavage was measured kinetically by spectrofluorimetry. Caspase 3/7 activity was determined as the slope of the resulting linear regressions and expressed in arbitrary fluorescence units per minute.

#### Assessment of the Mitochondrial membrane potential (MMP)

For detection of changes in the MMP at the single cell level, the lipophilic cation JC-1 was used as described elsewhere [14].

#### SubG1 DNA content and cell cycle distribution

For detection of cell cycle distribution, PI staining of DNA after fixation was used, as described elsewhere [14].

### Detection of Necrosis

#### Propidium Iodide (PI) uptake assay

The assay is based on the quantification of PI stained necrotic cells. Therefore, after compound exposure, PI (2 µg/ml) was added to each well of a 96-well microtiterplate for 5 min at RT under continuous shaking. Next, the plates were centrifuged at 189 g (3 min, RT). The amount of intracellular PI fluorescence was measured using a Tecan microplate reader at Ex: 525 nm/Em595 nm.

#### LDH release assay for tissue slices

The percentage of lactate dehydrogenase (LDH) release into the supernatant as surrogate for membrane integrity was determined using the LDH Mono-P assay (Analyticon, Lichtenfels, Germany) according to the manufacturer’s instructions. The amount of LDH being released into supernatant was determined and normalized to protein content of individual slices, which was determined by a Pierce BCA assay (Thermo Scientific, Dreieich, Germany).

### Isolation and Analysis of Side Population (SP) Cells

SP and non-SP cells were analysed and isolated from Huh7 cells as described before [22]. Briefly, Huh7 cells were trypsinized, counted and one million cells/ml resuspended in DMEM w/o phenol red containing 2% FCS and 10 mM HEPES. The cells were stained with 20 µg/ml Hoechst 33342 alone or together with verapamil (50 µM) at 37°C for 90 min. Then, the cells were washed once with ice cold HBSS containing 2% FCS and 10 mM HEPES. Analysis and sorting of SP and non-SP cells was performed using MoFlo (DAKOCytomation). Afterwards, cells were washed with HBSS, counted and seeded in culture dishes for another 24 h. Cells were then exposed to MTBITC and subsequently used for analysis.

### Detection of Aldehydedehydrogenase

Expression of the enzyme Aldehydedehydrogenase (ALDH) was analysed using the ALDEFLUOR Kit from Stemcell Technologies (Grenoble, France) according to manufacture’s protocol. As positive control, the ALDH inhibitor DEAB, which was provided in the kit, was used.

### Cell Migration (scratch) Assay

The scratch assay was performed as described elsewhere [23]. Light microscopic images acquired for each sample were analyzed quantitatively by using the computing software Image J (http://rsbweb.nih.gov/ij/index.html). For each image, distances between one side of scratch and the other was measured. By comparing the images from time 0 to the last time point (72 h) the distance of each scratch closure was obtained and the half-time for gab closure calculated.

### Cell Synchronisation by Serum Deprivation and DMSO

For cell synchronisation at G0/G1, the medium was removed from HepG2 cells and replaced by medium without FCS for 96 h. After that time, the cells were either exposed to DMSO or MTBITC for another 24 h in medium containing different FCS concentrations. The cells were then harvested and analysed for their DNA content distribution.

In a second set of experiments, 2% DMSO was added to the culture medium of adherent growing HepG2 cells for 72 h. Next, the cells were supplied with fresh culture medium containing either DMSO or MTBITC for 24 h, subsequently harvested and analysed for their DNA content distribution.

### *In vivo* Tolerability of MTBITC

Crl:NU-Foxn1mice (nude mice) were purchased from Charles River, Erkrath, Germany and obtained in microisolators in barrier conditions. Treatment was done by daily oral gavage with vehicle or MTBITC suspended in vehicle for 18 days.

### Gene Expression Analysis of p53 using Quantitative Real Time PCR

Total RNA was isolated with the RNeasy mini Isolation kit from Qiagen (Hilden, Germany), followed by a purification step using the RNase-Free DNase kit from Qiagen (Hilden, Germany). A total of 2 µg of RNA from each sample was employed for cDNA production using the First Strand cDNA Synthesis Kit from Fermentas (St. Leon-Rot, Germany). The cDNA samples were then diluted 1∶2 to 1∶5 and amplified with the LightCycler FastStart DNA Master^PLUS^ SYBR Green I Kit from Roche (Mannheim, Germany). p53 cDNA was amplified using sense 5′-CCTTCCCAGAAAACCTACCA-3′, and antisense 5′-TCATAG GGCACCACCACACT-3′ oligonucleotides which produce a 371 bp fragment. The reference gene beta-microglobulin cDNA was amplified using sense 5′-AGGCTATCC AGCGTACTCCA-3′ and antisense 5′-ACGGCAGGCATACTCATCTT-3′ oligonucleotides which produce a 248 bp fragment. All primer pairs cross intron/exon boundaries and thus, PCR products do not represent genomic DNA contamination. PCR conditions were: first 95°C for 10 min, followed by 40 cycles at 95°C for 10 s, 59°C for 10 s and 72°C for 30 s. Amplification products were quantified with the software Lightcycler 2.0 from Roche, Mannheim, Germany, by preparing a standard curve using known dilutions of the standard cDNA. To normalize the p53 mRNA expression for sample-to-sample differences in RNA input, RNA quality, and reverse transcriptase efficiency, the reference gene beta-microglobulin was amplified in parallel. All of the experiments were performed in triplicate.

### Gene Expression Analysis using a Signaling Pathway Array

RNA isolation from 10^6^ cells per sample was performed with the RT^2^ qPCR-Grade RNA Isolation Kit (SABioscience, Frederick, Maryland, USA). cDNA was synthesized using the RT^2^ PCR Array First Strand Synthesis Kit (SABioscience, Frederick, Maryland, USA). Quantitative real-time PCR of the human p53 signaling pathway RT^2^ Profiler™ Array (catalog no.: PAHS-027, SABioscience, Frederick, Maryland, USA) was done using a MyiQ Single-Color Real-Time PCR Detection System (Bio-Rad, München, Germany). For data analysis the web-based RT^2^ Profiler™ PCR Array Data Analysis was used. Data were analyzed by the ‘2^−ΔΔCt^ method’ using the software provided by the manufacturer. For each gene, fold-change was calculated as the difference in gene expression between two groups. The results were expressed as mean of 3 independent experiments.

### Protein Analysis by Immunoblotting

Protein concentration was determined as described by Bradford [24]. For immunoblotting, 20 µg of protein were mixed with ready made SDS-containing sample buffer, supplemented with 0.5% β-mercaptoethanol. Electrophoresis was performed according to the method of Laemmli [25]. The gel was then transferred to a nitrocellulose membrane by tank blotting. The unspecific binding sites were blocked with 5% low fat milk in TBS/T and incubated with primary, and subsequently horseradish peroxidase (HRP)-labeled secondary antibody for 1 h or over night. After antibody incubation, the proteins of interest were detected by chemiluminescence technique. A digital image of the western blot was captured by a CCD camera. Size approximations were taken by comparing the stained bands to that of a protein standard loaded during electrophoresis. The process was repeated for the structural protein β-actin.

### Silencing of p53 by RNAi

HepG2 cells were harvested by trypsination and 2.5×10^5^ cells were subjected to reverse transfection with Transpass R1 (no.: M2551S) from NEB (Frankfurt a. M., Germany). For transfection, 10 µl TransPass R1 were mixed with 240 µl DMEM and incubated for 15 min at RT before addition of siRNA oligomers (p53 ShortCut siRNA Mix, no.: N2011S, NEB, Frankfurt a. M., Germany; control siRNA cat.no. sc-37007, Santa Cruz Biotechnologies, Santa Cruz, USA; fluorescein conjugated control siRNA, no.: N2100S, NEB, Frankfurt a. M., Germany) at a final concentration of 25 nM, followed by another incubation step at RT for 15 min 250 µl of transfection complex were then transferred to each well and HepG2 cells containing DMEM +15% FCS w/o antibiotics were subsequently incubated with the complex for 24 h under standard cell culture conditions. The cells were then washed twice with PBS and supplemented with fresh DMEM containing 15% FCS for another 24 h before exposure to the test substance for 24 h.

### RT-MLPA and qPCR

RT-MLPA was used to analyze mRNA expression of Bcl-2 family members. RNA from HepG2 and Hep3B cells was isolated as described above. RT-MLPA (MRC Holland, kit RM002, R011-C1) was performed according to the manufacturer’s instructions. Briefly, specific mRNAs were reversely transcribed into cDNA and bound by two oligonucleotides consequently ligated by a heat-stable ligase. Each probe gives rise to an amplification product of unique length separated by capillary sequencer (Genescan). Analysis was performed with GeneMapper (Applied Biosystems). The sum of all peak data was set to 100% to normalize for fluctuations between different samples, and single peaks were calculated relative to 100%. Analysis of mRNA regulation of Bcl-2 and Bmf was not possible for technical reasons.

### Data Analysis

Results were calculated by using Graphpad Prism 5.0 software (La Jolla, CA). The median growth inhibitory concentration (IC50) value was calculated after normalization of the response and logarithmic transformation with the following equation: Y = 100/(1+10^∧^((LogIC50-X)*HillSlope)). Variance analysis between groups was performed by one-way ANOVA and significance of difference between control and treatment groups was analyzed using Dunnett multiple comparison test. The differences with p≤0.05 (*) or p≤0.01 (**) were considered statistically significant.

## Results

### Cytotoxicity of ITC in Liver Tumor Cells Compared to Normal Hepatocytes

**Figure 1:**
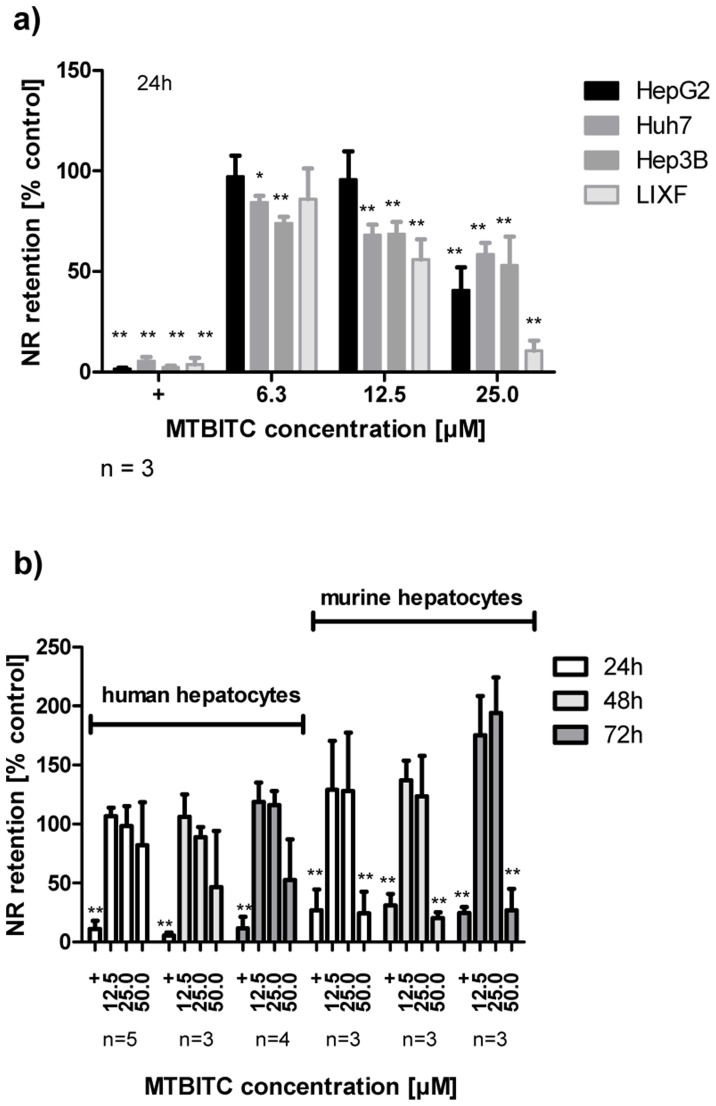
MTBITC selectively kills liver tumor cells. MTBITC treatment selectively attenuates cell survival of liver tumor cells (a) as compared to primary hepatocytes (b). Cell viability was assessed using the NR retention assay after exposure to MTBITC for 24–72 h. Results were expressed relative to control (0.1% DMSO), bars are mean+SD, (number of independent experiments is given below the figures). Positive control (+), 0.01% Triton X-100. ** ≤p<0.01 compared to DMSO.

We used the NR retention assay for assessment of MTBITC cytotoxicity on cancer cells in comparison to normal hepatocytes. This assay has been reported as high sensitive cytotoxicity parameter [26]. A concentration-dependent viability loss, as determined by impaired lysosomal NR retention capability was observed in all tested cancer cells after MTBITC treatment for 24 h (figure 1a). In contrast, in human hepatocytes, no relevant viability loss was seen within 24–72 h treated with <50 µM MTBITC (figure 1b). In murine hepatocytes, MTBITC triggered some processes enlarging the neutral-red accumulating capacity of lysosomes after 72 h of MTBITC treatment (figure 1b). Based on results derived after 24 h MTBTIC treatment, IC50 values were calculated at 23.18 µM (HepG2), 20.89 µM (Huh7), 31.97 µM (Hep3B), 13.11 µM (LIXF), 72.09 (human hepatocytes) and 47.81 (murine hepatocytes).

### Growth Arrest and Apoptosis Induction in Human HCC Cells and Normal Hepatocytes/PCLS

**Figure 2:**
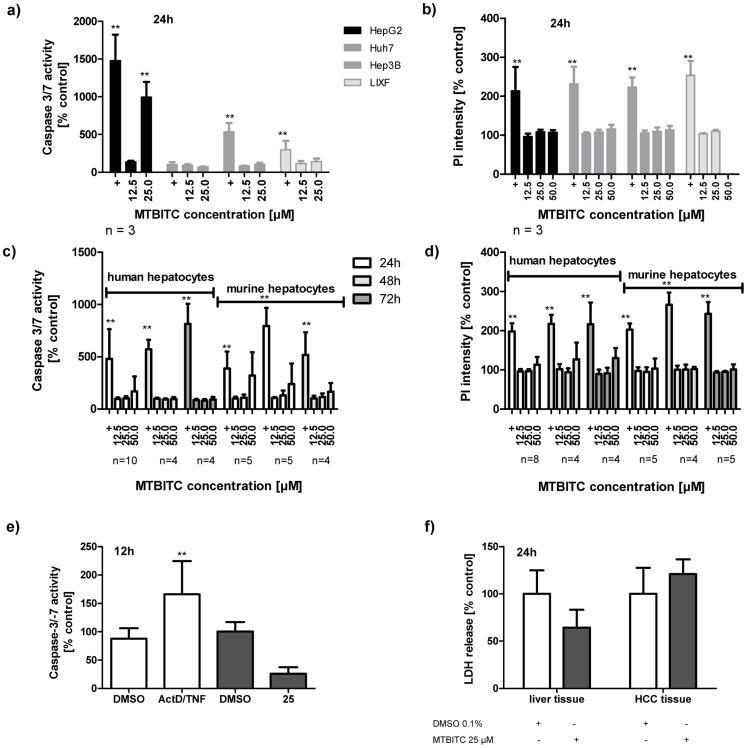
MTBITC selectively triggers apoptosis in liver tumor cells. Impact of MTBITC on apoptosis (a, c, and e) or necrosis (b, d and f) induction in malignant and healthy cells. Distinct apoptosis induction was assessed using caspase 3/7 activity after cell treatment with MTBITC for 12 to 72 h. Positive control (+): 10 µM CPT or staurosporine (a, c), 1 µg/ml actinomycin D/100 ng/ml TNF (ActD/TNF) (e). Necrosis induction was quantified using specific uptake of PI in isolated cells (b and d) or LDH release from PCLS (f). Positive control (+): 0.2% triton X-100. Results were expressed relative to the solvent (0.1% DMSO). Bars are mean+SD, (number of independent experiments is given below the figures; experiments conducted on PCLS were conducted in triplicate.).

**Figure 3:**
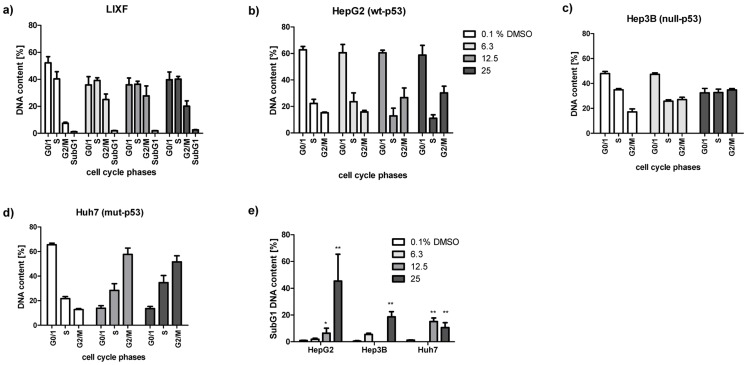
Cytostatic and cytotoxic effect of MTBITC on HCC cells. G2/M arrest (a to d) and apoptosis (e) induction after treatment of HCC cells with MTBITC, as determined by PI staining of DNA and flow cytometry analysis. Impact of MTBITC or 0.1% DMSO (solvent) was determined after 24 h (LIXF) or 72 h (HepG2, Huh7, Hep3B). Bars are mean+SD (n = 3).

To evaluate the nature of viability impairment observed in MTBITC-stressed HCC cells, we sought to establish whether this was due to a halt in cell growth, apoptotic or necrotic processes (figure 2 and 3). We first analyzed the cells for caspase 3/7 activation as specific apoptosis marker. Within 24 h, MTBITC induced significant apoptosis at 25 µM, but only in p53-wt (HepG2) cells; no increase could be seen in p53-mut (Huh7) cells, p53-null (Hep3B) cells or LIXF (figure 2a). Normal hepatocytes remained unaffected by the treatment, as investigated in human or murine cells at 24 h (figure 2c). We could not find any signs that MTBITC induced a necrotic reaction in malignant or normal cells, even at 50 µM, as determined by PI cell staining (figure 2b and d). Conditions of repeated exposure (up to 72 h), as would be the case under chemotherapy, did not further sensitize healthy hepatocytes to apoptosis (figure 2c) or necrosis (figure 2d). The observations made in normal hepatocytes were further verified using PCLS. This *ex vivo* model allows covering the complexity of liver structure and the multiplicity of metabolic, homeostatic, endocrine and biotransformation functions. Therefore it better reflects the high level of biological organization of the organ [27]. Exposure of PCLS to 25 µM MTBITC did not increase apoptosis (figure 2e) or necrosis (figure 2f) rate in healthy tissue but rather suppressed it, as compared to control slices. In contrast, the positive control, 1 µg/ml actinomycin D combined with 100 ng/ml TNF induced profound hepatic apoptosis (figure 2e).

We then measured the impact of MTBITC on cell cycle. The subG1 peak was thereby used as apoptosis indicator. After 24 h exposure to MTBITC, a strong concentration-dependent G2/M arrest was evident in LIXF (figure 3a) and all HCC cell lines tested but again without any clear signs of apoptosis in p53-mut or p53-null cells (data not shown). Then our analysis was extended to a 3-day period of MTBITC exposure, as we had already established that a prolonged MTBITC treatment did not increase damage to healthy cells. As shown in figure 3b-d, the G2/M block maintained under ITC treatment and this was in the order p53-null cells >p53-mut cells >p53-wt cells. Although within 24 h, p53-impaired cells did not undergo cell death, 72 h-treatment with MTBITC triggered apoptosis in all the cell lines tested (figure 3e).

### Effect of MTBITC on TIC Cells

**Figure 4:**
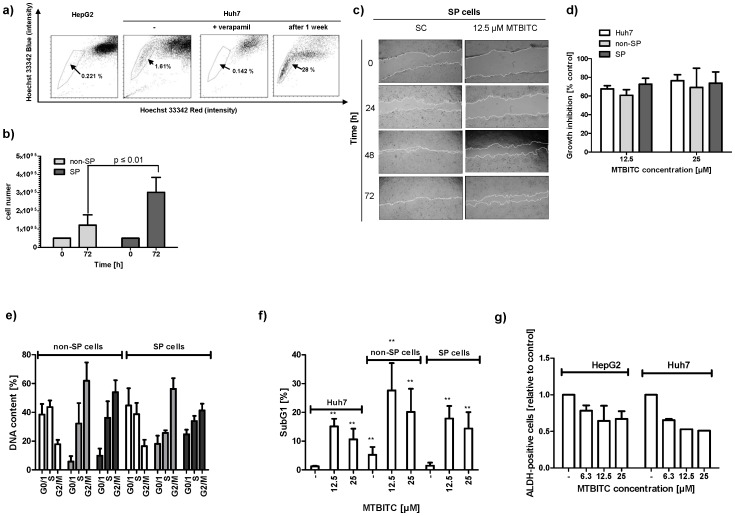
Sensitivity of TIC towards MTBITC. a) TIC were characterized by their capability to efflux Hoechst dye. Verapamil at 50 µM was used as positive control. Reanalysis of Hoechst dye efflux capability was done 7 days after sorting. For this SP cells were cultured in normal culture medium to reach the desired cell number. Then, the cultured SP cells were stained with Hoechst 33342 and analyzed by FACS. b) Cell growth of SP and NSP cells. Subpopulations were seeded at 50.000 cells/well in culture medium and cell number determined after 72 h using trypane blue staining. c) MTBITC reduces SP cell migration. Scratch assays in SP cells treated with solvent control, 12.5 µM MTBITC for 0 to 72 h. Microscopic pictures from one representative experiment out of three independent experiments are shown. d) Growth inhibition by MTBITC in Huh7, non-SP and SP subpopulations as determined by cell counting after 72 h. e) G2/M arrest and f) apoptosis induction after treatment of non-SP and SP cells with MTBITC, as determined by PI staining of DNA and flow cytometry analysis. Impact of MTBITC or 0.1% DMSO (solvent) was determined after 72 h. Bars are mean+SD (n = 3). g) MTBITC reduces ALDH-positive subpopulations in HepG2 and Huh7 cell lines. Cells were treated with MTBITC or 0.1% DMSO (−) for 24 h and subsequently ALDH activity was analysed by staining with ALDEFLUOR substrate and flow cytometry. Results were expressed relative to solvent control, bars are mean+SD, (HepG2, n = 3, Huh7 n = 1).

We next investigated the impact of MTBITC on chemoresistant SP cells, isolated from Huh7 cells using the DNA-binding dye Hoechst 33342 and flow cytometry. In contrast to the HepG2 cell line, which contained less than 0.3% of SP cells, the Huh7 cell line contained SP cells at a concentration of about 1.5% and was therefore used for further experiments (figure 4a). Appropriate SP discrimination in Huh7 cells was carried out by ABC transporter inhibition control experiments using verapamil (figure 4a), which successfully blocked Hoechst dye efflux. Characterization of the sorted cell populations showed that i) the capability of SP cells to efflux Hoechst dye is lost for the most part within one week in cell culture (figure 4a), as demonstrated by reanalysis of sorted SP cells. ii) SP cells grew significantly faster than NSP cells under the same treatment conditions, as determined after 72 (figure 4b). iii) Migration of SP cells was higher than that of total Huh7 cell population, assessed by the scratch assay (data not shown). Migration was also used as another parameter to investigate the chemotherapeutic potency of MTBITC. As shown in figure 4c, within 48 h, control cells had almost completely moved towards the opening of the newly created gab and closed the "scratch". In contrast, establishment of complete cell-cell contact was still not evident after 72 h in MTBITC treated cells. Determination of cell migration rate showed that under MTBITC treatment SP cells needed 44.1 h to close to gab by 50%, control cells 26.2 h. No drug sensitivity differences in terms of growth inhibition could be observed after MTBITC treatment between SP and NSP cells (figure 4d). Our further analysis revealed that MTBITC arrested non-SP as well as SP subfractions at G2/M and pushed them into programmed cell death, although to a less extent in SP cells as compared to their non-SP counterpart (figure 4e and f). We next investigated the ability of MTBITC to reduce the amount of ALDH-positive cells from HepG2 and Huh7 cell lines. As depicted in figure 4g, MTBITC-treatment concentration-dependently resulted in an abrogation of this marker; at 25 µM exposure for 24 h, ALDH-positive fraction was reduced by 30% (HepG2) and 50% (Huh7).

### Cell Cycle Dependency of MTBITC-induced Cytotoxicity and Apoptosis Induction in wt-p53 Cells

**Figure 5:**
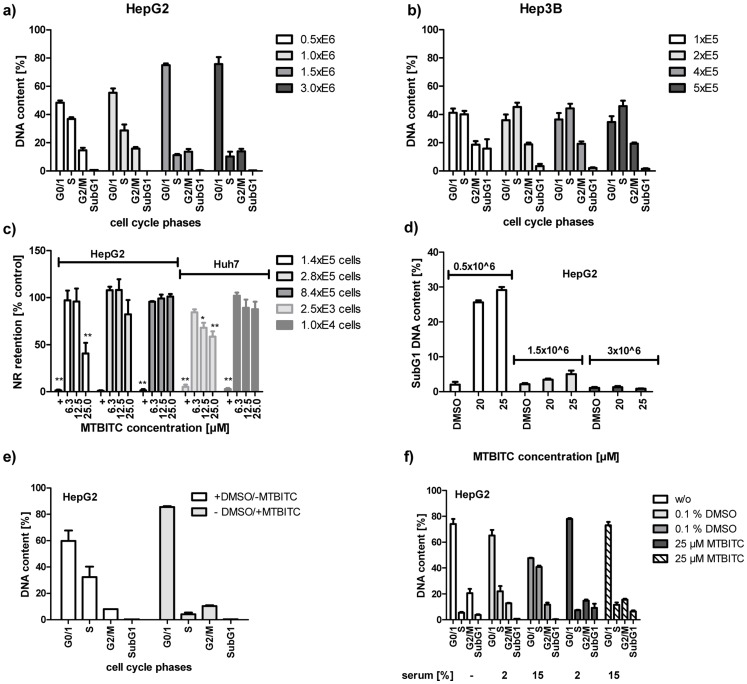
Cell cycle dependency of MTBITC-induced cytotoxicity and apoptosis induction in HCC cells. (a–d) influence of cell number on sensitivity to MTBITC-induced growth arrest and cytotoxicity, as assessed by DNA content analysis (a, b), neutral red retention (c) or subG1 peak analysis (d) of HCC cells. 0.1% DMSO was used as solvent control, 0.01% triton X-100 as positive control (+). (e and f) impact of G0/1 phase synchronization on cell sensitivity to MTBITC in HepG2 cells, as determined by DNA content analysis. (e) cells were treated with 2% DMSO for 96 h and subsequently treated with 25 µM MTBITC or 0.1% DMSO for another 24 h. (f) cells were starved for 96 h and subsequently treated with 25 µM MTBITC or 0.1% DMSO for another 24 h, while adding varying amounts of serum. Bars are mean+SD, (n = 3).

In contrast to p53-null (Hep3B) cells, use of p53-wt (HepG2) cells at low densities was necessary to ensure that experiments could be completed before cell–cell contact inhibited proliferation and cells arrested at G0/G1 (figure 5a and b). Furthermore, this reduced proliferation capacity of p53-wt cells then dramatically impacted the cytotoxic potency of MTBITC (figure 5c). A comparable effect was seen for p53-mut cells (figure 5c), but not p53-null cells (data not shown). Apoptosis induction, as assessed at 24 h, was consequently lost when wt-p53 cells were exposed to the ITC at increasing densities (figure 5d). This observation raised the possibility that MTBITC induced cell death was signalled only distal to G0/G1. To investigate this further, we used phase synchronized p53-wt cells, achieved by serum deprivation. As seen in figure 4e, termination of a temporary serum withdrawal caused the cells to re-enter normal cell cycle. When treated with a combination of serum and MTBITC, G0/G1 cells were prevented from re-entry into cell cycle but so was the onset of apoptosis. The same effect was observed when 2% DMSO was used to force cells into G0/G1 (figure 5f).

### The Relevance of TP53 Death Pathway Activation in wt-p53 Cells by MTBITC

**Figure 6:**
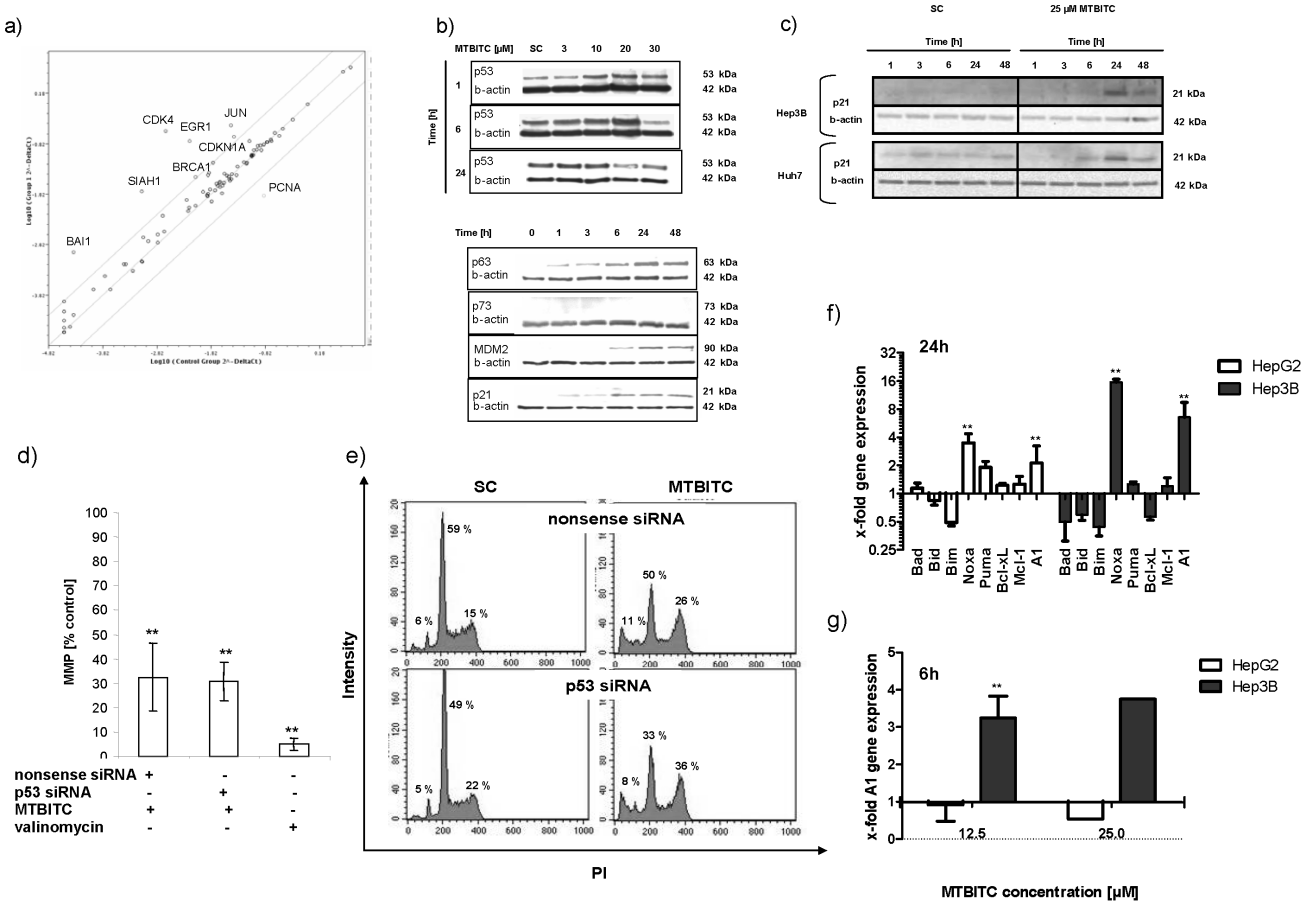
Relevance of p53 pathway activation in MTBITC-triggered growth arrest. (a) gene expression analysis of wt-p53 (HepG2) mRNA using real - time PCR arrays. The scatter blot compares expression of 84 p53 pathway genes between solvent and 25 µM MTBITC treated (3 h) cells. The solid diagonal line represents no change in expression. Any data point above the upper line represents genes that are upregulated >4-fold. Any data point below the lower line represents genes that are downregulated >4-fold. Values are mean of three independent experiments. Immunoblotting of p53 pathway related proteins (b) or p21 (c) after exposure to MTBITC or solvent to HCC cells. β-actin was used as loading control. (d and e) silencing of p53 in wt-p53 (HepG2) cells using RNAi. HepG2 cells were reverse transfected with p53-RNAi or nonsense RNAi for 24 h and then exposed to MTBITC or solvent for another 24 h. Impact of p53-RNAi on MTBITC-induced mitochondrial membrane potential collapse (MMP) (d) or G2/M arrest (e). Bars are mean ± SD, (n = 3). (f and g) RT-MLPA mRNA expression analysis of pro- and anti-apoptotic Bcl-2 family members in MTBITC-treated HCC cells, bars are mean+SD. (n = 3). All results in figure 5 were calculated relative to the solvent control (SC, 0.1% DMSO).

We were as yet unable to conclusively clarify the necessity of p53 for cell death induction. So, we subsequently used a pathway gene array to determine whether early cell death observed in wt-p53 cells could be due to the upstream activation of p53-controlled genes. As shown in figure 6a, within 3 h seven genes were up regulated by more than 4-fold, while one was down regulated (see also table S1). We could not detect any regulation of p53 on a gene level, however p53 protein was strongly accumulated in a concentration-dependent manner (figure 6b). p63 shares similar transcriptional functions with p53, including its potential for inducing apoptosis and growth suppression, although with varying efficiency [28]. With respect to this similarity between p53 and p63, the influence of MTBITC-treatment on the modulation of p63 was additionally investigated. A time-dependent increase of p63 protein content could be observed after 1-h treatment with MTBITC (figure 6b). p73 was undetectable in p53-wt cells (figure 6b). Furthermore, a time-dependent increase in MDM2 protein level could be detected in cells exposed to MTBITC which is in accordance with the change in p53 protein expression and explain very well its observed time-dependent variation (figure 6b). The transcriptional activation of p21, a major target for transactivation by p53 and crucial player in mediating growth arrest when DNA damaging agents are present, was found to be upregulated by gene array analysis (i. e. CDKN1A) and also confirmed by western blotting (figure 6a). When mut-p53 (Huh7) as well as null-p53 (Hep3B) cells were investigated for p21 expression, the same time dependent induction could be observed which indicated a p53-independent regulation of p21 (figure 6c).

Although these results confirmed TP53 pathway activation in wt-p53 cells, their relevance for MTBITC-triggered signalling into growth arrest and cell death was still unclear. Therefore, in a next step, we used RNA interference and studied the effects of p53 silencing on cell cycle distribution and apoptosis. These parameters were assessed by JC-1 staining of mitochondria using flow cytometry (figure 6d) and DNA content analysis (figure 6e). A nonsense siRNA was used as control. Efficient p53 silencing after 24 h (70%) was confirmed by quantitative PCR (data not shown). We found that the absence of p53 did not affect the outcome of MTBITC efficacy on HCC cells. Combined treatment of cells with MTBITC and p53 siRNA neither sensitized nor protected the cells from MTBITC-induced apoptosis or growth arrest as compared to nonsense siRNA-treated cells (see figure 6d and e).

### Involvement of the Bcl-2 Family in MTBITC-triggered Cell Death

We then focused on the intrinsic apoptosis pathway which is regulated mainly by the Bcl-2 family. Interestingly, amongst all pro-apoptotic BH3-only genes, known to act as cell stress-sensors, only Noxa was up-regulated significantly both in wt-p53 (HepG2) and null-p53 (Hep3B) cells (figure 6f). At least in Hep3B cells, Noxa is induced in an p53-independent manner. In HepG2 cells, mRNA induction of Noxa upon MTBITC treatment was dose-dependent (1.7-fold at 12.5 µM vs. 3.5-fold at 25 µM) (fig. 6f and data not shown) and could already be observed after 6 h of treatment (data not shown). In contrast, in Hep3B cells, Noxa mRNA was only up regulated upon MTBITC treatment at the high dose (fig. 6f). Moreover, in parallel, levels of the anti-apoptotic Bcl-2 protein A1, an important Noxa antagonist, strongly increased in these cells (fig. 6g). No regulation of the downstream pro-apoptotic Bcl-2 proteins Bax or Bak was observed upon MTBITC treatment in both cell lines.

Together, all these experiments validated the idea that the cytostatic/cytotoxic effects induced by MTBITC were indeed caused by a mechanism independent of p53 function. Thus, these results eliminated the possibility that endogenous p53 is critical for a potential therapeutic effect of MTBITC on liver cancer cells.

### *In vivo* Tolerability of MTBITC

**Figure 7:**
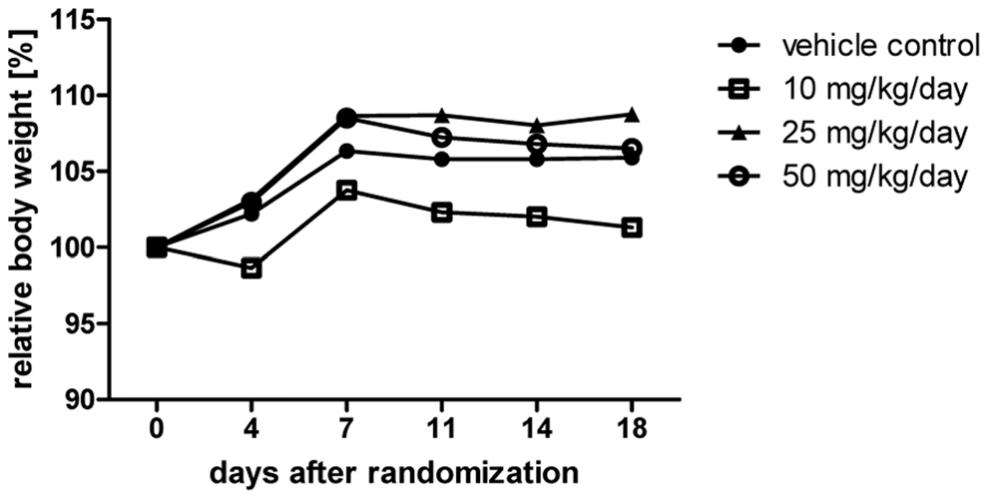
*In vivo* tolerability of MTBITC. Mice were randomized into groups and treated for 18 days by daily gavage with vehicle or MTBITC at 10, 25 or 50 mg/kg/d. Time course of relative mean body weight is given in the figure. The body weight was set at day 0 (start of gavage) to 100% for each group and plotted for data gathered at days 4, 7, 11, 14 and 18. n = 3 each group.

Based on our results derived *in vitro* we then investigated the tolerability of orally applied MTBITC *in vivo*. Body weight determined over a treatment period of 18 days was used as parameter for agent-induced cytotoxicity. As shown in figure 7, orally applied MTBITC did not result in relevant body-weight loss.

## Discussion

ITC have attracted a great deal of attention not only because of their cancer preventive [29] but also because of their potential therapeutic action [30]. Although available data are promising, so far *in vitro* experiments on the therapeutic efficacy of ITC mostly consider their action on cancer cells without taking into account the risk of unwanted effects on the healthy tissues of the same target organ. Our data now demonstrate that MTBITC is capable of killing liver cancer cells, irrespective of the tumor suppressor p53 status. It has been suggested that in some cell types loss of a wt-p53 genotype renders cells resistant to the lethal effects of DNA damage induced by radiotherapy or genotoxic agents [31], [32]. Others demonstrated that irradiation-induced cell death was only delayed in p53-null in contrast to wt cells [33], [34]. For ITC, the role of p53 in the cytotoxic/cytostatic response of cancer cells has been controversial up to now. For instance, sulforaphane (SFN) the oxidized metabolite of MTBITC formed *in vivo* [35], led to a clear p53 protein accumulation in human leukaemia cells [36]. In contrast it remained unchanged in the colorectal carcinoma cell line HT-29 [37]. Our data now clearly show that loss of p53 function has only a temporal effect on the initiation of cell death, delaying rather than inhibiting. The fact that in Hep3B cells, A1 mRNA was induced much stronger than in HepG2 cells (6.6-fold vs. 2.1-fold) indicates that the Noxa-A1 balance may be involved in the higher sensitivity of HepG2 than Hep3B cells for apoptosis induction by MTBITC. Particularly the early up regulation of A1 preceding Noxa in Hep3B cells may explain the delayed apoptosis induction in Hep3B. This is in line with a study conducted with SFN in LNCaP prostate carcinoma cells [38] and led us to the conclusion that p53 is not essential for ITC-triggered liver tumor cell destruction. As mutations in the p53 gene are among the most common alterations observed in HCC (approximately 30% [39] to 50% [40] of HCC contain a mutated or inactivated p53) this finding is quite interesting with regard to further research on the general applicability of ITC as cancer therapeutic compounds.

In our study we could show that the cytotoxic action of MTBITC works even with chemoresistant subpopulations of liver cancer cells. This was recognized either by the successful reduction of ALDH activity level in subpopulations of HepG2 or Huh7 cells or inhibition of drug transporter-upregulated (SP) cells from Huh7. Both characteristics are significant determinants of cell drug resistance. It is thought that due to their ability to detoxify many potentially cytotoxic chemicals, chemotherapy fails after several cycles, although the tumor was responsive to the first cycles [41]. Lately, SFN was also demonstrated to inhibit the self-renewal of breast cancer stem cells using reduction of ALDH-positive cell populations as marker [10]. Furthermore, using the same marker for TIC, this ITC was successful against chemoresistant cells of the prostate and pancreas, either alone or in combination with other chemotherapeutic agents such as gemcitabine or sorafenib [11], [42]–[44]. Interestingly, SFN preferentially targeted breast cancer stem cells instead of bulk cell population [10]. In contrast, in our study, no such preference could be observed as growth inhibition and apoptosis induction by MTBITC were not significantly different between the populations using Hoechst dye efflux as TIC marker.

Therapeutic selectivity is one of the most important considerations in cancer chemotherapy. The present data imply that MTBITC exerts a tumor cell-selective cytotoxicity. So far, the specific cytotoxicity of ITC to cancer cells has been almost exclusively hypothesized based on results from transformed and non-transformed cell lines. As an example, differentiated HT29 human colon carcinoma cells were less sensitive to allyl ITC, BITC or PEITC as compared to the de-differentiated cells [45], [46]. SFN was also shown to be non-cytotoxic to differentiated colon carcinoma (CaCo2) cells, although it killed the undifferentiated counterpart [47]. In the non-malignant, sub confluent proliferating rat liver epithelial cell line RL-4, apoptosis was induced at 25 µM AITC-glutathione conjugate; in proliferating RL-34 rat hepatocytes, BITC induced apoptosis at a concentration of 20 µM [48], [49]. These cell lines express a variety of differentiation markers that are typical of the normal phenotype; however, they remain immortalized cells that proliferate continuously. Normal hepatocytes are highly differentiated and therefore non-dividing cells. During our experiments we ensured confluence to avoid any dedifferentiation processes. Most chemotherapeutic agents preferentially affect proliferating cells and our results suggest that this also applies for ITC. In our study, wt-p53 cells were not acutely affected by MTBITC when contact inhibition occurred or G0/G1 arrest was enforced by serum starvation or DMSO treatment. However, MTBITC still inhibited quiescent cells to re-enter the cell cycle. This fact should be considered with regard to proliferating hepatocytes or oval cells e. g. during regeneration processes of the organ. Of course besides proliferation, ITC have been shown to interfere with many factors that are altered in cancer cells. In hepatoma cells, interaction with the Bcl-2 family complex was shown by us in the present study, as well as modification of the intracellular signal cascades, regulating proliferation and apoptosis. Furthermore, a recent proteomic investigation on ITC interaction sites revealed that ITC have strong affinities for covalent binding to tubulin, specifically cysteines [50]. In general, binding of ITC to cellular thiol-residues, initially intracellular GSH, is a common feature. The affinity thereby strongly depends on ITC side chain and lipophilicity [51]. As a consequence, GSH depletion by ITC is associated with sensitivity to growth arrest and death in all cancer cells studied so far. However, HCC cells contain at least twice the amount of GSH as compared to normal hepatocytes [52]. Therefore, the relevance for this mechanism seems very questionable for interpretation of the present results. A further interesting explanation is provided by Clarke *et al.* [35]. These authors demonstrated that SFN treatment selectively decreased HDAC activity, increased acetylated histone H3 at the promoter for P21. This in turn induced p21 expression and increased tubulin acetylation in prostate cancer cells. The effect was connected to an elevated cytotoxic response of PC3 compared to non-malignant cells. In our study p21 expression was induced by MTBITC in all HCC cell lines under investigation. We therefore suggest this mechanism in HCC compared to normal hepatocytes to be studied more closely in a sequel study.

### Conclusions

In conclusion, we could demonstrate that MTBITC selectively targets liver tumor cells and their chemoresistant subpopulations. This observed cytostatic/cytotoxic potency is independent of TP53. As orally MTBITC treatment was tolerated by mice very well in our study, we suggest further evaluating its usage against liver cancer.

## Supporting Information

Table S1 Relative gene expression (fold change >4) in HepG2 cells by 3 h MTBITC-exposure compared to control.
(DOC)
