# Supplementary material for: Preclinical Evaluation of 4-Methylthiobutyl Isothiocyanate on Liver Cancer and Cancer Stem Cells with Different p53 Status
Source: PLoS One. 2013 Aug 2;8(8):e70846. doi: 10.1371/journal.pone.0070846 (PMC3732292; doi:10.1371/journal.pone.0070846)
Supplement: Table S1 — Relative gene expression (fold change >4) in HepG2 cells by 3 h MTBITC-exposure compared to control. (DOC) [file pone.0070846.s001.doc]

**Table S1: Relative gene expression (fold change > 4) in HepG2 cells by 3 h MTBITC-exposure compared to control**

| **Gene name** | **Reference sequence** | **Description** | **Fold change** | **p value** |
| --- | --- | --- | --- | --- |
| BAI | NM_001702 | Brain-specific angiogenesis inhibitor 1 | 24.82 | 0.0468 |
| BRCA1 | NM_007294 | Breast cancer 1, early onset | 4.19 | 0.2202 |
| CDK4 | NM_000075 | Cyclin-dependent kinase 4 | 123.52 | 0.0564 |
| CDKN1A | NM_000389 | Cyclin-dependent kinase inhibitor 1A (p21, Cip1) | 5.24 | 0.7825 |
| EGR1 | NM_001964 | Early growth response 1 | 28.09 | 0.0010 |
| JUN | NM_002228 | Jun proto-oncogene | 10.09 | 0.0030 |
| SIAH1 | NM_003031 | Seven in absentia homolog 1 (Drosophila) | 21.63 | 0.4278 |
| PCNA | NM_182649 | Proliferating cell nuclear antigen | -9.91 | 0.2449 |

(n =3)
